# Resistance Exercise and Art Therapy on Body Image in Breast Cancer: A Scoping Review

**DOI:** 10.1089/whr.2020.0058

**Published:** 2020-09-28

**Authors:** Corrie J. Effa, Naomi D. Dolgoy, Margaret L. McNeely

**Affiliations:** ^1^ Department of Physical Therapy, Faculty of Rehabilitation Medicine, University of Alberta, Edmonton, Canada.; ^2^ Department of Oncology, Cross Cancer Institute, University of Alberta, Edmonton, Canada.

**Keywords:** art therapy, body image, breast cancer, resistance exercise

## Abstract

***Background:*** Treatments for breast cancer are invasive, causing visible changes such as loss of the breast, body weight change, and hair loss. These changes in conjunction with the pressure for women to conform to societal beauty standards may lead to body image disturbance in breast cancer survivors (BCS). The aims of this scoping review were to explore the nature, characteristics, and extent of the literature examining resistance exercise or art therapy on body image in BCS; and examine how body image is defined and measured across the studies.

***Methods:*** We searched the literature up to January 2020, which included conducting electronic searches of three major databases and checking references of screened articles.

***Results:*** Ninety-three articles were identified, 28 underwent full-text screening, with 8 studies eligible for inclusion in the review. Five randomized control trials, one hybrid effectiveness-implementation trial, and two single group studies were found. All studies showed significant within-group difference in body image scores, with two studies showing a between-group difference in favor of resistance exercise. No studies were found combining resistance exercise and art therapy. None of the studies defined the aspect of body image they wished to measure, and only one used theory to inform their research.

***Discussion:*** Preliminary evidence supports the benefit of resistance exercise and art therapy as single interventions to improve body image perception among BCS. Findings suggest the need for closer attention to the delivery format of interventions. Future research is needed that is theory-informed, with a clear definition of the aspect of body image of interest, and with body image as the primary outcome.

## Introduction

### Breast cancer and body image

Breast cancer is the third-most frequently diagnosed cancer in Canada, and it is estimated that 27,400 Canadians will be diagnosed with the disease in 2020.^1^ Treatments for breast cancer are invasive, often resulting in visible changes in physical appearance, which can lead to body image concerns and psychological distress in breast cancer survivors (BCS).^2,3^

Treatment of breast cancer typically involves surgery, followed by radiation, chemotherapy, and hormonal treatments.^2^ Surgery involves removing part of the breast (lumpectomy), the entire affected breast (mastectomy), or both breasts (double mastectomy), which may cause BCS to feel mutilated, resulting in an immediate negative impact on their body image.^2^ Moreover, amputation of a breast is often associated with pain, swelling in the breast or limb (lymphedema), and changes in sensation to the breast and chest wall.^4^ As a result, the BCS may feel less feminine, less sexually attractive, and experience increased anxiety and depression.^2^

Radiation therapy is an adjuvant treatment involving high doses of radiation to destroy any remaining cancer cells in the region of the primary cancer.^5^ Chemotherapy is a common systemic treatment that uses pharmaceutical agents to kill cancer cells, reducing the risk of cancer returning or spreading.^5^ Physical effects that occur from radiation and chemotherapy treatment include weight gain, hair loss, skin irritation, skin discoloration, and hot flashes from early-onset menopause.^6,7^ Hormonal treatment—which is a treatment that blocks hormones to slow the growth of cancer cells—has also been known to impact sexual dysfunction.^7^

Many BCS experience profound fatigue from treatment, which can limit the social and physical activities they engage in.^8^ The compounding effect of the diagnosis, adjusting to the negative effects of cancer treatments on their bodies, and the impact of fatigue on everyday activities may be overwhelming for the BCS, reducing their overall quality of life.^9^

### Resistance exercise training and art therapy on body image

Both resistance exercise training (RET) and art therapy have the potential to improve body image concerns among BCS. Current research points to the benefits of RET on body image perception among the adult general population.^10^ While the mechanism of change is still unclear, there is evidence to suggest that for women, both subjective perceptions (self-efficacy, confidence) and objective improvements in fitness play a role in improving body image.^11^

Art therapy aims to influence the mind and body, which can be used to promote health and well-being.^12^ Current research within oncology points to art therapy as an intervention that creates space for self-expression that may be otherwise too painful to verbalize.^13^ In addition, art therapy can be used as a means for cancer survivors to redefine their priorities and personal identity so that they are more involved in self-care practices.^12^

### Context and purpose

While there is evidence to show that RET positively influences body image in the general population and that art therapy has improved psychological outcomes among cancer survivors, more research is needed to determine specifically if body image perception improves among BCS.

The potential of combining RET and art therapy came from the authors' experience at Wellspring Edmonton—a nonprofit center that offers supportive programs to meet the psychological, emotional, and educational needs of individuals and families living with cancer in Edmonton, Canada. Anecdotally, we noticed that BCS taking part in our RET program twice weekly, along with an art therapy class hosted at the center, appeared to report fewer body image-related concerns when compared with BCS participating in RET alone.

The purpose of this scoping review was to explore the research on RET and art therapy alone, and in combination as interventions to address body image in BCS. The aims of this review were to: (1) explore the nature, characteristics, and extent of the literature examining RET or art therapy on body image in BCS; and (2) examine how body image is defined and measured across the studies.

## Materials and Methods

A scoping review was performed to explore the literature broadly. The methodology utilized was based on the five-step procedure created by Arksey and O'Malley.^14^

A librarian was consulted at the University of Alberta to help identify relevant studies, through an effective search strategy. The searches were run on the databases CINAHL, PsycINFO, and Medline up to, and including January 2020 (see Appendix A1 for details).

Articles were considered eligible if they involved women with breast cancer in both the intervention and control groups (if they had a control group), involved RET or art therapy interventions, included body image as a primary or secondary outcome, and were available in English with accessible full texts. Articles were excluded if they were review articles, involved single case studies, or involved qualitative research with a sample size of 2 or less. Title and abstract screening were completed by one author (C.J.E.), and full-text screenings were completed by two authors (C.J.E. and M.L.M.). When conflicts were identified, the authors (C.J.E. and M.L.M.) achieved consensus through discussion.

Upon selecting the articles, we abstracted the following elements: author, year, country; study design and sample size; objectives related to body image; intervention details; program design; the body image tools used; fitness testing measured (if applicable); and study results; and any other considerations reported by study authors.

For collating, summarizing, and reporting the details, we examined the type of study, the intervention approach, and the chosen outcome measures. We further expanded on the concepts surrounding body image relevant to the following: whether the study involved body image as a primary outcome; how the study defined body image; any theory or theoretical framework used to inform the research; the rationale or justification for the intervention; data supporting intervention fidelity; whether the study reported or controlled for confounding factors such as age, surgery and other cancer treatments, timing since diagnosis, body mass index, and lymphedema. These listed concepts allowed us to further explore the nature of the body image studies in BCS.

## Scoping Review Results

The search resulted in 115 articles. Once duplicates were removed, 93 articles were included for initial screening. Of the 93 articles, 28 were deemed potentially eligible and were selected for full-text review. Following formal screening, seven articles met all inclusion criteria.

One study had a primary objective of self-esteem as measured by the physical self-perception profile (PSPP).^15^ As the PSPP has questions related to “attractive body,” the study was included in the review.^15^

**FIG. 1.**
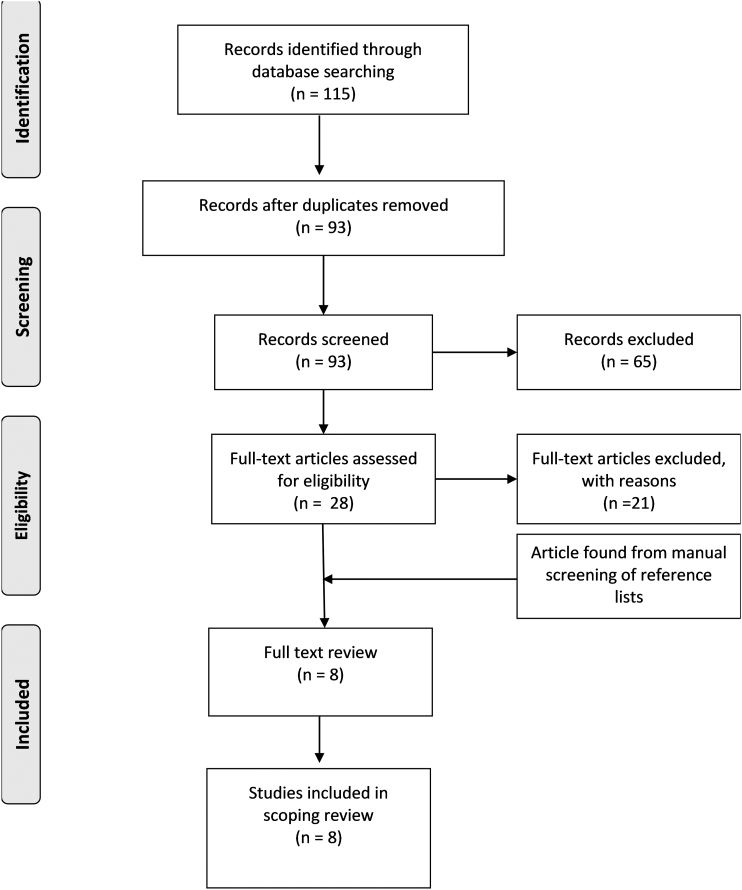
Study flow.

The reference lists of the seven articles were reviewed, which identified an additional study, for a total of eight articles (Fig. 1). Of the eight articles, seven examined the effect of RET alone,^15–21^ and one made use of art therapy alone as an intervention.^22^ No studies were found combining both RET and art therapy.

### Study design

Of the seven eligible articles involving RET, a variety of study designs were used. Four were randomized control trials (RCTs),^15,16,19,21^ one was a hybrid effectiveness-implementation trial,^17^ and two were nonrandomized trials.^18,20^ Sample sizes ranged from 15 to 234. The single art therapy study was an RCT with a sample size of 41.^22^ Body image was a primary outcome in two RET studies,^16,18^ and a secondary outcome in the other six studies.^15,17,19–22^ See Table 1 for a description of the included studies.

**Table 1.** Characteristics of the Included Studies

Study/country	Study design sample size	Objectives related to body image	Intervention details	Program design—individual or group	Body image tool used and fitness measures	Study results	Considerations
Speck et al.^16^/United States	• RCT *n* = 234 BCS with or without lymphedema • Treatment group *n* = 113 • Control group *n* = 121	Examine the effects of weight training on body image in PAL trial (primary objective)	• 1-year weight-lifting 2 × /week at the YMCA 13 weeks supervised and then the rest unsupervised • Control group on a 1-year waiting list	Small groups (2–6 people) for first 3 months. Individual and unsupervised for remaining 9	• BIRS • Baseline and 12 months • Anthropometry, 1-RM bench and leg press • Strength measurements: 10-RM chest press and 1-RM leg press	• Significant between-group difference in BIRS by 12% after 1 year in favor of intervention group compared with 2% increase in the control group (*p* < 0.0001) • Significant improvements in 1-RM bench press and leg press (*p* < 0.0001) after 1-year • No significant change in QOL	• Also measured general QOL (SF-36) • No association observed between lymphedema status and lower BIRS values
Beidas et al.^17^/United States	Hybrid type 1 effectiveness-implementation trial *n* = 84. With or without lymphedema	• Determine if modifying the PAL a trial would still prove to be effective and safe for survivors • Qualitatively assess barriers and facilitators to implementing this design by interviewing key stakeholders	4 small group training sessions led by a physiotherapist and 2 × /week strength training at home	4 supervised small-group training sessions, the remaining unsupervised individual	• BIRS • 1-RM bench and leg press	• Significant within-group improvement in body image from baseline to 12 months (*p* < 0.001) • Significant within-group improvement in muscular strength (*p* < 0.001)	Used the same measures as the PAL trial
Benton et al.^18^/United States	• Nonrandomized 8-week trial *n* = 20 • *n* = 12, YRT • *n* = 8, ORT	To evaluate the effect of age on changes in body image and overall QOL after a resistance training program (primary objective)	Both YRT and ORT groups 2 × /week for 8 weeks identical supervised resistance training sessions	Individualized supervised exercise program	• BIRS • Arm-curl test, 30-second chair-stand test, 10-RM bench press, 1-RM leg press	• Significant within-group improvement in BIRS total scores (*p* < 0.001) • Significant between-group difference in BIRS total scores, with a greater improvement in the YRT group compared with the ORT group (*p* < 0.01) • Within-group improvements in strength and function (*p* < 0.001)	• The program brought awareness to functional decline in older participants but was too short in duration to show improvements and benefits of being active • The ORT group was less susceptible to noticing improvements in fitness compared with the YRT group
Musanti et al.^15^/United States	• Prospective RCT 12-weeh duration with or without lymphedema • Flexibility *n* = 12 • Aerobic *n* = 10 • Resistance *n* = 9 • Aerobic and resistance *n* = 11	To determine which exercise modality has the most significant impact on self-esteem (attractive body is a component measured)	Participants were assigned to a home-based 12-week exercise program A 3 × /week, R 3 × /week, AR 4–5 × /week, or F as the control	Individualized home-based exercise program	• PSPP measure of self-esteem has a component measuring attractive body • Submaximal Bruce protocol treadmill test, 6-RM chest press and leg press, YMCA bench press, and shoulder/hip flexibility	• Significant within-group improvement in attractive body in Resistance group only (*p* < 0.000) • Significant within-group improvements in muscular strength (chest press *p* = 0.032 and curls *p* = 0.013 in Resistance, Aerobic and Resistance, and Flexibility groups)	• Did not report between-group differences for body image scores • Other measures include: Rosenberg Self-Esteem Scale, Piper Fatigue Scale, and Hospital Anxiety and Depression Scale to measure fatigue and mood
Paulo et al.^19^/Brazil	• RCT *n* = 36 postmenopausal women between ages 50–80 years • Resistance and aerobic group *n* = 18 • Control group *n* = 18	Evaluate exercise program on QOL of BCS aged 50–80 years undergoing aromatase inhibitor therapy (body image is a component of QOL)	• Supervised resistance and aerobic exercise 3 × /week for 9 months and health education session 1 × /month • Control group invited to participate in stretching and relaxation 2 × /week for 45 minutes for 9 months	Supervised group exercise	EORTC QLQ-BR23	• Significant within-group improvement in body image in both exercise and control groups after 3 months compared with baseline (*p* < 0.001) and after 6 months compared with 3 months	• Used other QOL measures (EORTC QLQ-C30, SF-36) • Did not measure physical changes in fitness
Stan et al.^20^/United States	Prospective, interventional, one-arm, open-label study *n* = 15 with or without lymphedema	• Assess feasibility of Pilates exercises after mastectomy (primary outcome). • Changes in shoulder range of motion, neck flexibility, posture, lymphedema, height, QOL, mood, and body image (secondary outcome)	36 45-minute sessions of Pilates mat classes over a 12-week period	Participants could choose to come into the exercise facility and do a group class or do a DVD at home	MBSRQ	• Statistically significant within-group improvements in health evaluation (*p* = 0.049) and body area satisfaction (*p* = 0.017) • Shoulder mobility significantly improved in abduction (*p* = 0.002) and internal rotation (*p* = 0.028)	
Eyigor et al.^21^/Turkey	• RCT *n* = 52 without lymphedema • Hospital exercise program *n* = 27 • Control group home exercise program *n* = 25	Determine the effect Pilates has on BCS' functional capacity, flexibility, depression and QOL (body image as a component of QOL)	• Supervised Pilates exercise 1 hour 3 × /week for 8 weeks • Control group was instructed to do home exercises that were given to everyone in a handout	Group Pilates class	• EORTC QLQ-BR23 • 6 MWT, and modified sit and reach test	• Significant within-group improvement in body image as BR23 functional scores significantly improved in exercise group from pre- to postvalues (*p* < 0.05) • Significant between-group improvement in 6 MWT in exercise group compared with control (*p* = 0.00)	Additional psychological measures: Beck Depression Test, Brief Fatigue Index, EORTC QLQ-30
Svensk et al.^22^/Sweden	• RCT *n* = 41 • Art therapy *n* = 20 • Control group *n* = 20	Evaluate art therapy intervention on self-rated QOL among BCS (body image as a component of QOL)	• Randomized into art therapy 1 × /week for 5 weeks or control group, which had no art therapy • QOL assessments before intervention, 2 and 6 months later	Individual sessions with art therapist	EORTC QLQ-BR23	• Significant within-group improvement in art therapy group for body image between baseline and 6-month measurement (*p* = 0.027)	• Additional Measures: QOL measures: WHOQOL-BREF (Swedish version)

AR, aerobic and resistance exercise; BCS, breast cancer survivors; BIRS, Body Image and Relationship Scale; DVD, digital versatile disc; EORTC QLQ-BR23, European Organization for Research and Treatment-QOL questionnaire and breast cancer-specific module; MBSRQ, Multidimensional Body-Self Relations Questionnaire; MWT, minute walk test; ORT, ages 60-80 years group; PAL, physical activity and lymphedema; PSPP, physical self-perception profile; QOL, quality of life; RCT, randomized control trial; RM, repetition maximum; SF, Short Form Health Survey Questionnaire; WHOQOL-BREF, World Health Organization Quality of Life Instrument; YMCA, Young Men's Christian Association; YRT, ages 40–59 years group.

### Intervention details: RET

The RET interventions involved either 2 to 3 sessions per week, with the length of program ranging from 8 weeks to 1 year. Programs varied considerably, with one home exercise program,^15^ two supervised group sessions,^19,21^ one supervised one-on-one sessions,^18^ one study with a combination of home and group sessions,^20^ and two studies with a combination of supervised small-group sessions and individual unsupervised sessions.^16,17^ The control groups were either wait-list control or followed an intervention involving walking or flexibility training.

The exercise programs all had a component of strength training; however, the chosen intervention protocol varied considerably between studies. Two of the studies did Pilates exercise,^20,21^ four studies used free weights, weight machines, or a combination of the 2,^16–19^ and one study used resistance bands.^15^

### Intervention details: art therapy

The art therapy study involved 1 hour of supervised one-on-one sessions per week for 5 weeks.^22^ The individual art therapy sessions were led by one of two art therapists.^22^ The following supplies were available at each session: paper, oil pastel and paints, paintbrushes tempera fluid, pencils, charcoal, tape, and scissors.^22^

### Defining body image and corresponding outcome measures

None of the eight studies defined body image nor provided detail of what aspect of body image they were aiming to measure (Table 2). Of the eight studies, the instruments chosen to measure body image varied substantially, with four different scales used: The Body Image and Relationships Scale (BIRS), the European Organization for Research and Treatment-QOL questionnaire and breast cancer specific module (EORTC QLQ-BR23), Multidimensional Body-Self Relations Questionnaire (MBSRQ), and the PSPP.

**Table 2.** Body Image Characteristics of the Included Studies

	Primary outcome	Body image definition	Theoretically informed	Rationale/justification for intervention	Intervention fidelity	Control of confounders
**Defining parameters**	Whether body image was considered a primary outcome of the study	Whether the study defined body image, or explicitly stated what aspect of body image planning to measure, aside from talking about the tool	Whether the study used theory to explain mechanism of change in body image perception in breast cancer	Whether the study provided justification and rationale for conducting the intervention, highlighting the gap in literature	Whether the control group was comparable with the intervention, the appropriate measurements administered, and adherence to protocol	Whether the study controlled for the following confounding variables: lymphedema, age, BMI, time since surgery, and treatment type
Study						
Speck et al.^16^	+	−	−	+	+	+
Beidas et al.^17^	−	−	−	+	+	−
Benton et al.^18^	+	−	−	+	+	−
Musanti et al.^15^	−	−	+	+	+	−
Paulo et al.^19^	−	−	−	+	−	−
Stan et al.^20^	−	−	−	+	+	−
Eyigor et al.^21^	−	−	−	+	−	−
Svensk et al.^22^	−	−	−	+	+	−

+ Present in the article according to parameters set.

− Lacking in the article according to parameters set.

BMI, body mass index.

Three studies used the BIRS, which has components of strength and health, social barriers and appearance, and sexuality.^16–18^ The BIRS is a disease-specific measure, where the questions are specific to the changes and potential issues that BCS face.^23^ The tool has 32 items, where five responses are possible (1 = disagree strongly to 5 = agree strongly).^23^ The higher the score, the greater the number of issues the BCS experience.^23^

Three studies,^18,20,21^ including the art therapy article, used one of EORTC QLQ-BR23, which has questions related to body image, sexual functioning, sexual enjoyment, and future perspective.^24^ Similar to the BIRS, this 23-item tool is also a disease-specific measure, where five responses are possible, ranging from 0 (not at all) to 4 (very much).^24^ Scoring higher on the symptom-oriented questions indicates increased symptoms, and scoring higher on the functioning scales represents higher levels of functioning.^24^

One study used the MBSRQ,^20^ which measures attitudes related to evaluative, cognitive, and behavioral components of body image.^25^ The full version of this tool is 69 items, consisting of 10 subscales.^25^ One unique aspect of this tool is that it has a section measuring one's attitude toward fitness and health, which is advantageous for those doing RET interventions.^25^ While the tool was designed for adults and adolescents aged 15 years or older, it is yet to be validated for breast cancer.^26^

One study used the PSPP,^15^ which measures four subdomains of self-esteem: perceived body attractiveness, sport competence, physical strength, and physical conditioning.^27^ The respondents select one of two opposing statements that they relate to the most and consequently choose if it is “sort of me” or “really true of me.”^15^ The attractive body component was the component relevant for this review. This tool has also not yet been validated for BCS.^15^

### Use of theory

Upon reviewing all studies, only one article was theoretically informed (Table 2).^15^ The Self-Esteem Model was applied to help explain the connections between the exercise modality and physical self-esteem in BCS.^15^

### Summary of findings: RET

All of the RET studies using the BIRS tool showed statistically significant improvements in overall scores within the intervention group.^16–18^ Speck and colleagues' article was one of the two articles to report a significant between-group difference in the BIRS in favor of the intervention group (12% improvement) when compared with the control group (2% improvement) at 12 months.^16^

The second article, Benton and colleagues, had two groups doing the same exercise intervention—one with younger BCS (40–59 years) and the other with older cancer survivors (60–80 years).^18^ While both groups showed significant improvement on the BIRS after the intervention, the younger group demonstrated a significantly larger improvement compared with the older group, suggesting that age may be a factor in the response to RET in terms of body image perception.^18^

The remaining studies in this review reported significant within-group improvements only.^15,19–21^ Musanti et al. reported significant improvements in the “attractive body” component of the PSPP from baseline to postintervention in the RET group.^15^ Similarly, Stan et al. showed a significant improvement in the “health evaluation” and “body area satisfaction” subscales of the MBSRQ only.^20^ Finally, Eyigor et al. showed significant within-group improvements in body image in the EORTC QLQ-BR23 “functional” score category, while the “symptom” category remained unchanged.^21^

The four RET studies that measured muscular strength before and after the intervention all showed significant improvements in strength.^15–18^ One study did not include fitness testing as an outcome in its protocol, which was the primary reason for not meeting the intervention fidelity requirement in Table 2.^19^

### Summary of findings: art therapy

The art therapy trial showed significant improvements in quality of life in the intervention group compared with the control group, however, there were no clinically significant differences between groups for body image as measured by the EORTC QLQ-BR23.^22^ Both the art therapy and control groups were found to improve in body image; however, the only statistically significant improvement in body image was found in the art therapy group at the 6-month follow-up.^22^

## Discussion

### Nature, characteristics, and extent of the literature

**FIG. 2.**
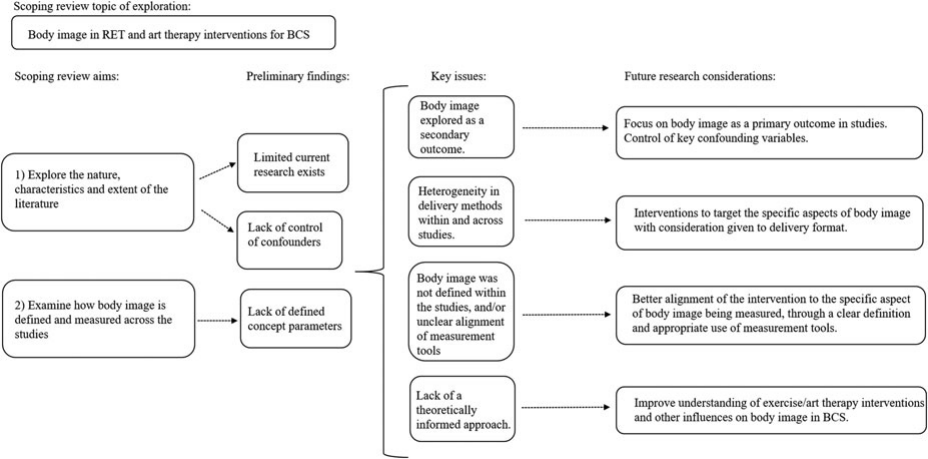
Key considerations of body image in RET and art therapy interventions for BCS. BCS, breast cancer survivors; RET, resistance exercise training.

While this scoping review provides preliminary evidence that RET can influence body image in BCS, continued research is indicated to evaluate the effectiveness of art therapy, as only one study, with a small sample size, was found. Although the studies provided adequate rationale and intervention fidelity for carrying out their respective interventions, several key issues arose in our scoping review. These issues, along with resulting research considerations from our two aims, are depicted in Figure 2.

To start, only one of the five RCT articles showed a significant between-group difference in body image scores,^16^ while the remaining four reported a within-group difference.^15,19,21,22^ Svensk et al. posit that improvements in body image may be related to the “response shift” phenomenon.^22^ This phenomenon occurs when the cancer survivors recalibrate their personal values and internal standards over time as they cope with their disease, thus reporting lower instances of distress or concern despite their symptoms remaining unchanged.^28^ Consequently, the within-group improvements may have resulted, in part, or entirety from this phenomenon, rather than due to the intervention itself.

Studying the response shift phenomenon in body image among BCS is complex, as in the context of surgery alone, the shift depends on body image investment before diagnosis, the type of surgery, and postsurgical complications.^29,30^ Further RCTs are needed to control for this phenomenon, and to better evaluate the effectiveness of the intervention.

Most of the studies in this review included body image as a secondary outcome, resulting in less control of potential confounding variables; as baseline body image levels were not considered in the study inclusion/exclusion criteria, which may influence the results of body image outcomes. In addition, only one study controlled for the surgery type,^22^ and only two studies controlled for time since surgery,^15,16^ despite the fact that both the type of surgery and time since surgery are known to impact BCS' body image perception.^29,31^

One study examined the impact of age on body image in BCS participating in RET^18^ and only one study controlled for age.^16^ Similar to studies in the general population, further research is needed to determine if RET is helpful in improving body image among older women.^10^

The study by Speck and colleagues controlled for the greatest number of confounding variables, and serves as an example for future studies.^16^

### Delivery formats

This scoping review found a wide range in exercise formats (*i.e.*, group vs. individual; home vs. community) both across and within studies, providing a challenge to determine whether one delivery method was more effective than another. The authors of one study hypothesized that improvements in body image may have resulted from the group delivery format, concluding that the group environment provided opportunities for the BCS to share their experiences, without fear of feeling judged.^19^

Similarly, research among the general population suggests the need for a placebo control group with a matched delivery format consistent with the intervention group.^10^ Among the RET articles in this review, two of the seven articles used a wait-list control or had control participants follow an individualized home exercise program when the experimental intervention was delivered to participants in a group format.^16,21^

Ensuring that both the intervention and control groups have equal amounts of attention and group interaction is paramount in understanding the effect of the delivery format on outcomes. Thus, future research should consider not only the intervention but also the delivery format.

### Defining body image

Another significant finding of our review was that none of the studies defined body image, providing a challenge for the reader to understand the rationale for the intervention and choice in measurement tools. Similar to quality of life, body image affects the cognitive, physical, emotional, social, and behavioral health of the BCS, demonstrating the multidimensionality of the construct.^32^ As such, specifically defining the aspect of body image of interest—whether it be all aspects or in the realm of positive body image psychology, subjective satisfaction, perceptual, affective, cognitive, or behavioral components of body image—will provide clarity on the selection of the measurement tool.^32^

For example, the MBSRQ measures appearance satisfaction (an aspect of body image defined as the affective perceptions individuals have about their body)^33^ and also measures appearance investment (which is a separate dimension that is not correlated with appearance satisfaction).^32^ Appearance investment does not measure body image but rather measures how important physical appearance is to the individual, and whether cognition and actions are centered around physical appearance.^32^

Along with a definition, future studies should consider alignment of the definition, study objectives, and the chosen measurement tool, to facilitate better understanding of the meaning of the results.^32^ To inform future research in the field, Table 3 lists key measurement tools and the aspect of body image being measured.

**Table 3.** Body Image Tools and the Corresponding Aspect of Body Image Measured

Body image tool	Type of instrument	Aspect of body image measured	Summary of the tool
BIRS	Global satisfaction measure specific to breast cancer	Self-perceptions of appearance, health, physical strength, sexuality, relationships, and social functioning^16–18^	• Breast cancer specific • A global view of how body image affects psychological adjustment and functioning in daily life
PSPP	Physical self-esteem	Measures perceptions of body attractiveness^15^	• Appearance satisfaction/dissatisfaction • Whether a person views the body as attractive
EORTC QLQ-BR23	Quality-of-life measure specific to breast cancer	5 Scales: Body image, sexual functioning, sexual enjoyment, future perspective (functional scales), arm symptoms, breast symptoms, upset due to hair loss, and systematic side effects (symptom scales)^19,21,22^	• Breast cancer specific • Based on ability to cope with changes from treatment
MBSRQ	Global satisfaction measure	Measures appearance evaluation, appearance orientation, fitness evaluation, fitness orientation, health evaluation, health orientation^20^	Body image as a reflection of: • affective elements (feelings toward body); • cognitive elements (thoughts and awareness toward the body); • behavioral elements (behaviors connected toward the body)

EORTC QLQ-BR23, European Organization for Research and Treatment of Cancer-QOL questionnaire and breast cancer specific module.

### Lack of theory-informed research

Finally, the use of theory was lacking in the studies included this review, as only one study reported using theory to inform their research.^15^ A recent systematic review examining the efficacy of psychosocial and physical activity-based interventions to improve body image among BCS similarly concluded that the lack of theory-informed research limits the understanding of the potential mechanisms behind the findings.^3^ This issue is not unique to breast cancer studies, but is also seen with research involving the general population.^10^

The benefit of using theory-informed research will not only help guide hypothesized causal pathways between the intervention (RET or art therapy) and the outcome measure (body image), but also facilitates consideration of the behavioral, social, and subjective implications of the construct.^34^

### Limitations

There are several limitations of this scoping review that need to be highlighted. Only three databases were searched extensively, and as a result, some articles may have been missed. However, the three databases were carefully selected with the help of a librarian to ensure that the searches were relevant and comprehensive.

Despite the extensive search, only one study was found examining art therapy and no studies were found examining RET in combination with art therapy.

The findings are further limited by the wide variability in the chosen assessment tools, objectives, and intervention protocols limiting our ability to make clear recommendations on the benefit of RET and art therapy on body image.

## Conclusions

This review provides preliminary evidence showing that engaging in health-promoting activities—such as art or resistance exercise—has the potential to improve body image among BCS.

To improve the quality of research examining body image in BCS, consideration should be given to the following: (1) incorporating body image as a primary outcome to better control confounding variables; (2) attending to the details of the intervention itself, with focus on delivery formats; (3) defining the aspect of body image that is of interest, and selecting the assessment tool that aligns best with the stated objectives; and (4) using a theory-informed approach as a means to understand the influences of the RET or art therapy intervention on body image.

We propose that adopting these recommendations will progress our understanding of supportive interventions such as RET and art therapy on body image in BCS, potentially enhancing BCS wellness outcomes and quality of life.

## Abbreviations Used

BCS: breast cancer survivors
BIRS: Body Image and Relationships Scale
BMI: body mass index
EORTC QLQ-BR23: European Organization for Research and Treatment-QOL questionnaire and breast cancer specific module
MBSRQ: Multidimensional Body-Self Relations Questionnaire
MWT: minute walk test
PAL: physical activity and lymphedema
PSPP: physical self-perception profile
QOL: quality of life
RCTs: randomized control trials
RET: resistance exercise training
RM: repetition maximum

## Appendix

### CINAHL

1. (MH “Breast Neoplasms”) or breast cancer
2. (MH “Body Image+”) or (MH “Body Image Disturbance (NANDA)” or (MH “Body Image Enhancement (Iowa NIC)”) or (MH “Body Image Disturbance (Saba CCC)”) or (MH “Body Image (Iowa NOC)”)
3. (self esteem or body image or body representation or self image) or ((perception* or thought* or attitude* or feeling* or belief*) n5 (body or bodies or appearance))
4. ((MH “Art Therapy”) or (MH “Canadian Art Therapy Association”) or (MH “Art Therapy (Iowa NIC)”) or art* therap*
5. (MH “Muscle Strengthening+”) OR ((Resist* exercise* or weight train* or resist* training or strength train* or weightlifting or (lift* n4 weight*) or gravity resistive or isotonic or isometric))
6. 1 AND (2 OR 3) AND (4 OR 5)

### PsycINFO

1. breast neoplasms/or neoplasms/or mammography/or mastectomy/
2. breast.mp. [mp = title, abstract, heading word, table of contents, key concepts, original title, tests and measures]
3. mammary.mp. [mp = title, abstract, heading word, table of contents, key concepts, original title, tests and measures]
4. 2 or 3
5. (cancer* or oncolog* or carcinoma* or tumor* or tumour* or neoplasm* or metasta* or malignan*).mp. [mp = title, abstract, heading word, table of contents, key concepts, original title, tests and measures]
6. 4 and 5
7. 1 or 6 [Breast cancer]
8. body image/or body image disturbances/or body awareness/
9. body image*.mp. [mp = title, abstract, heading word, table of contents, key concepts, original title, tests and measures]
10. self-esteem/or self-concept/or self-confidence/or self-perception/
11. self esteem.mp. [mp = title, abstract, heading word, table of contents, key concepts, original title, tests and measures]
12. ((perception* or thought* or attitude* or feeling* or belief*) adj5 (body or bodies or appearance)).mp.
13. 8 or 9 or 10 or 11 or 12 [Body image]
14. art therap*.mp. [mp = title, abstract, heading word, table of contents, key concepts, original title, tests and measures]
15. art therapy/or creative arts therapy/
16. weightlifting/
17. (Resist* exercise* or weight train* or resist* training or strength train* or weightlifting or (lift* adj4 weight*) or gravity resistive or isotonic or isometric).mp.
18. 14 or 15 or 16 or 17 [Exercise or art therapy]
19. 7 and 13 and 18

### Medline

1. breast neoplasms/or breast carcinoma in situ/or breast neoplasms, male/or carcinoma, ductal, breast/or carcinoma, lobular/or inflammatory breast neoplasms/or unilateral breast neoplasms/or triple negative breast neoplasms/
2. breast.mp.
3. mammary.mp. [mp = title, abstract, original title, name of substance word, subject heading word, floating sub-heading word, keyword heading word, protocol supplementary concept word, rare disease supplementary concept word, unique identifier, synonyms]
4. 2 or 3
5. (cancer* or oncolog* or carcinoma* or tumor* or tumour* or neoplasm* or metasta* or malignan*).mp.
6. 4 and 5
7. 1 or 6 [Breast Cancer]
8. Body Image/
9. Body image*.mp. [mp = title, abstract, original title, name of substance word, subject heading word, floating sub-heading word, keyword heading word, protocol supplementary concept word, rare disease supplementary concept word, unique identifier, synonyms]
10. Body representation*.mp. [mp = title, abstract, original title, name of substance word, subject heading word, floating sub-heading word, keyword heading word, protocol supplementary concept word, rare disease supplementary concept word, unique identifier, synonyms]
11. Self-image.mp. [mp = title, abstract, original title, name of substance word, subject heading word, floating sub-heading word, keyword heading word, protocol supplementary concept word, rare disease supplementary concept word, unique identifier, synonyms]
12. Self-esteem.mp. [mp = title, abstract, original title, name of substance word, subject heading word, floating sub-heading word, keyword heading word, protocol supplementary concept word, rare disease supplementary concept word, unique identifier, synonyms]
13. ((perception* or thought* or attitude* or feeling* or belief*) adj5 (body or bodies or appearance)).mp.
14. 8 or 9 or 10 or 11 or 12 or 13 [Body Image]
15. Art Therapy/
16. art therap*.mp.
17. Resistance Training/or Weight Lifting/
18. (Resist* exercise* or weight train* or resist* training or strength train* or weightlifting or (lift* adj4 weight*) or gravity resistive or isotonic or isometric).mp.
19. 15 or 16 or 17 or 18 [Exercise or Art Therapy]
20. 7 and 14 and 19

## References

[1] Canadian Cancer Society Statistics 2020. Canadian Cancer Society. Available at: https://www.cancer.ca/en/cancer-information/cancer-type/breast/statistics/?region=on Accessed 525, 2020

[2] Kunkel EJ, Chen EI, Okunlola TB Psychosocial concerns of women with breast cancer. Primary Care Update for Ob Gyns 2002;9:129–134

[3] Lewis-Smith H, Diedrichs P, Rumsey N, Harcourt D Efficacy of psychosocial and physical activity-based interventions to improve body image among women treated for breast cancer: A systematic review. Psychooncology 2018;27:2687–2699 30161285 10.1002/pon.4870

[4] Langellier KM, Sullivan CF Breast talk in breast cancer narratives. Qual Health Res 1998;8:76–94 10558328 10.1177/104973239800800106

[5] Aebi S, Davidson T, Gruber G, Castiglione M, Group EGW Primary breast cancer: ESMO Clinical Practice Guidelines for diagnosis, treatment and follow-up. Ann Oncol 2010;21(Suppl. 5):v9–v14 20555111 10.1093/annonc/mdq159

[6] Coates A, Abraham S, Fox RM, et al. On the receiving end-patient perception of the side-effects of cancer chemotherapy. Eur J Cancer Clin Oncol 1983;19:203–208 6681766 10.1016/0277-5379(83)90418-2

[7] Dizon DS, Suzin D, McIlvenna S Sexual health as a survivorship issue for female cancer survivors. Oncologist 2014;19:202–210 24396051 10.1634/theoncologist.2013-0302 PMC3926787

[8] Bower JE, Ganz PA, Desmond KA, Rowland JH, Meyerowitz BE, Belin TR Fatigue in breast cancer survivors: Occurrence, correlates, and impact on quality of life. J Clin Oncol 2000;18:743–753 10673515 10.1200/JCO.2000.18.4.743

[9] Brunet J, Sabiston CM, Burke S Surviving breast cancer: Women's experiences with their changed bodies. Body Image 2013;10:344–351 23490552 10.1016/j.bodyim.2013.02.002

[10] SantaBarbara NJ, Whitworth JW, Ciccolo JT A systematic review of the effects of resistance training on body image. J Strength and Cond Res 2017;31:2880–2888 28723817 10.1519/JSC.0000000000002135

[11] Martin Ginis KA, Eng JJ, Arbour KP, Hartman JW, Phillips SM Mind over muscle? Sex differences in the relationship between body image change and subjective and objective physical changes following a 12-week strength-training program. Body Image 2005;2:363–372 18089201 10.1016/j.bodyim.2005.08.003

[12] Wood MJ, Molassiotis A, Payne S What research evidence is there for the use of art therapy in the management of symptoms in adults with cancer? A systematic review. Psychooncology 2011;20:135–145 20878827 10.1002/pon.1722

[13] Reynolds F, Prior S The role of art-making in identity maintenance: Case studies of people living with cancer. Eur J Cancer Care 2006;15:333–341 10.1111/j.1365-2354.2006.00663.x 16968314

[14] Arksey H, O'Malley L Scoping studies: Towards a methodological framework. Int J Soc Res Methodol 2005;8:19–32

[15] Musanti R A study of exercise modality and physical self-esteem in breast cancer survivors. Med Sci Sports Exerc 2012;44:352–361 21796050 10.1249/MSS.0b013e31822cb5f2

[16] Speck RM, Gross CR, Hormes JM, et al. Changes in the body image and relationship scale following a one-year strength training trial for breast cancer survivors with or at risk for lymphedema. Breast Cancer Res Treat 2010;121:421–430 19771507 10.1007/s10549-009-0550-7

[17] Beidas RS, Paciotti B, Barg F, et al. A hybrid effectiveness-implementation trial of an evidence-based exercise intervention for breast cancer survivors. J Natl Cancer Inst Monogr 2014;2014:338–345 25749601 10.1093/jncimonographs/lgu033 PMC4411538

[18] Benton MJ, Schlairet MC, Gibson DR Change in quality of life among breast cancer survivors after resistance training: Is there an effect of age? J Aging Phys Act 2014;22:178–185 23579251 10.1123/japa.2012-0227

[19] Paulo TRS, Rossi FE, Viezel J, et al. The impact of an exercise program on quality of life in older breast cancer survivors undergoing aromatase inhibitor therapy: A randomized controlled trial. Health Qual Life Outcomes 2019;17:1–12 30658629 10.1186/s12955-019-1090-4 PMC6339353

[20] Stan DL, Rausch SM, Sundt K, et al. Pilates for breast cancer survivors: Impact on physical parameters and quality of life after mastectomy. Clin J of Oncol Nurs 2012;16:131–141 22459522 10.1188/12.CJON.131-141

[21] Eyigor S, Karapolat H, Yesil H, Uslu R, Durmaz B Effects of pilates exercises on functional capacity, flexibility, fatigue, depression and quality of life in female breast cancer patients: A randomized controlled study. Eur J Phys Rehabil Med 2010;46:481–487 21224783

[22] Svensk AC, ÖSter I, Thyme KE, et al. Art therapy improves experienced quality of life among women undergoing treatment for breast cancer: A randomized controlled study. Eur J Cancer Care 2009;18:69–77 10.1111/j.1365-2354.2008.00952.x 19473224

[23] Hormes JM, Lytle LA, Gross CR, Ahmed RL, Troxel AB, Schmitz KH The body image and relationships scale: Development and validation of a measure of body image in female breast cancer survivors. J Clin Oncol 2008;26:1269–1274 18323550 10.1200/JCO.2007.14.2661

[24] Sprangers MA, Groenvold M, Arraras J, et al. The European Organization for Research and Treatment of Cancer breast cancer-specific quality-of-life questionnaire module: First results from a three-country field study. J Clin Oncol 1996;14:2756–2768 8874337 10.1200/JCO.1996.14.10.2756

[25] Cash TF 2002. Body Image Assessments: MBSRQ. Available at: www.body-images.com/assessments/mbsrq.html Accessed 529, 2020

[26] Rhondali W, Chisholm GB, Filbet M, et al. Screening for body image dissatisfaction in patients with advanced cancer: A pilot study. J Palliat Med 2015;18:151–156 25188590 10.1089/jpm.2013.0588 PMC4308823

[27] Fox K, Corbin C The physical self-perception profile: Development and preliminary validation. J Sport Exerc Psychol 1989;11:408–430

[28] Dabakuyo TS, Guillemin F, Conroy T, et al. Response shift effects on measuring post-operative quality of life among breast cancer patients: A multicenter cohort study. Qual Life Rese 2013;22:1–11 10.1007/s11136-012-0135-5 22383104

[29] Collins KK, Liu Y, Schootman M, et al. Effects of breast cancer surgery and surgical side effects on body image over time. Breast Cancer Res Treat 2011;126:167–176 20686836 10.1007/s10549-010-1077-7 PMC3265936

[30] Carver CS, Pozo-Kaderman C, Price AA, et al. Concern about aspects of body image and adjustment to early stage breast cancer. Psychosom Med 1998;60:168–174 9560865 10.1097/00006842-199803000-00010

[31] Fang SY, Shu BC, Chang YJ The effect of breast reconstruction surgery on body image among women after mastectomy: A meta-analysis. Breast Cancer Res Treat 2013;137:13–21 23225142 10.1007/s10549-012-2349-1

[32] Thompson JK The (mis)measurement of body image: Ten strategies to improve assessment for applied and research purposes. Body Image 2004;1:7–14 18089137 10.1016/S1740-1445(03)00004-4

[33] White CA Body image dimensions and cancer: A heuristic cognitive behavioural model. Psychooncology 2000;9:183–192 10871714 10.1002/1099-1611(200005/06)9:3<183::aid-pon446>3.0.co;2-l

[34] Hardeman W, Sutton S, Griffin S, et al. A causal modelling approach to the development of theory-based behaviour change programmes for trial evaluation. Health Educ Res 2005;20:676–687 15781446 10.1093/her/cyh022

